# A Systematic Review of Methods for Handling Missing Variance Data in Meta-Analyses of Interventions in Type 2 Diabetes Mellitus

**DOI:** 10.1371/journal.pone.0164827

**Published:** 2016-10-17

**Authors:** Sarah Batson, Hannah Burton

**Affiliations:** DRG Abacus, Bicester, United Kingdom; University of British Columbia, CANADA

## Abstract

**Aims:**

Meta-analysis is of critical importance to decision makers to assess the comparative efficacy and safety of interventions and is integral to health technology assessment. A major problem for the meta-analysis of continuous outcomes is that associated variance data are not consistently reported in trial publications. The omission of studies from a meta-analysis due to incomplete reporting may introduce bias. The objectives of this study are to summarise and describe the methods used for handling missing variance data in meta-analyses in populations with type 2 diabetes mellitus (T2DM).

**Methods:**

Electronic databases, Embase, MEDLINE, and the Cochrane Library (accessed June 2015), were systematically searched to identify meta-analyses of interventions in patients with T2DM. Eligible studies included those which analysed the change in HbA1c from baseline.

**Results:**

Sixty-seven publications reporting on meta-analyses of change in HbA1c from baseline in T2DM were identified. Approaches for dealing with missing variance data were reported in 41% of publications and included algebraic calculation, trial-level imputation, and no imputation.

**Conclusions:**

Meta-analysis publications typically fail to report standardised approaches for dealing with missing variance data. While no particular imputation method is favoured, authors are discouraged from using a no-imputation approach. Instead, authors are encouraged to explore different approaches using sensitivity analyses and to improve the quality of reporting by documenting the methods used to deal with missing variance data.

## Introduction

Meta-analysis is of critical importance for decision makers to assess the comparative efficacy and safety of interventions and is becoming an increasingly important component of health technology assessment. Indirect comparison and network meta-analyses are used to assess the comparative efficacy and safety of interventions in the absence of randomised controlled trials (RCTs) comparing these interventions. The most common method for analysing continuous outcomes in meta-analysis is to base the analysis on the mean change from baseline and an appropriate level of uncertainty such as the standard deviation (SD) [1]. A major problem for the meta-analysis of continuous outcomes is that trial publications do not consistently report the SDs for change in outcomes from baseline [2]. There is no empirical evidence exploring the reasons for missing SDs in RCTs but previous studies have reported that greater than 90% of studies report incomplete efficacy outcomes [3, 4]. In addition, these studies concluded that statistically significant trial efficacy results have statistically significantly higher odds of being fully reported in trial publications compared with non-significant results [3, 4]. The association of incomplete reporting of outcomes with statistical significance is of concern for meta-analysis. The omission of studies from a meta-analysis due to incomplete reporting may introduce bias and reduce statistical power.

The Cochrane handbook outlines methods of algebraic calculations for estimating the SD using alternative parametric summary statistics (p-values, confidence intervals [CIs], standard errors [SEs]) which may be reported in trial publications instead [5]. Algebraic calculation methods rely on reported parametric summary statistics with the assumption that they have approximate distributions. The statistical significance of hypothesis tests may not be reported as an exact value but instead in various ways, such as an upper bound or critical p-value. In practice a critical or upper-bound p-value (i.e. p<0.01) may be used as an approximation to compute the SD. This represents a conservative approach to quantifying uncertainty [5]. In practice many primary study publications fail to report any adequate measures of uncertainty around the change from baseline of continuous outcomes [3, 4] and, therefore, alternative methods for handling missing variance data are required.

Although it is recommended that authors are contacted for missing data, in practice they rarely respond. In the event that no measures of uncertainty are reported there are a number of methods for variance imputations reported in the literature [5–10]. A previous systematic review by Wiebe et al. highlighted that missing SDs is a widespread problem and that there is a lack of standardisation in the methods for handling missing variance data in meta-analysis [10]. This is likely a result of the lack of clear guidance in the Cochrane handbook or NICE technical support documents regarding the ‘gold standard’ approach to imputation.

Meta-analyses for type 2 diabetes mellitus (T2DM) are of particular interest because of the rising prevalence of the disease and the increasing burden on global healthcare resources. The demand for meta-analyses of diabetes therapies has grown over recent years due to the emergence of novel agents. The primary measure of control in T2DM is the change in HbA1c levels from baseline, which represents a relevant continuous outcome. This study aims to present a current review of the methods used to address missing SDs in meta-analysis of continuous outcomes in T2DM.

## Methods

### Search strategy

Electronic databases were searched on 4 June 2015 (Embase; MEDLINE^®^ In-Process & Other Non-Indexed Citations; Ovid MEDLINE; Cochrane Library [Cochrane Central Register of Controlled Trials; Cochrane Database of Systematic Reviews; Database of Abstracts of Reviews of Effects; Cochrane Library Health Technology Assessment]) [S1 Table, supporting information]. Two reviewers independently screened the titles and abstracts of identified citations using pre-specified eligibility criteria; potentially relevant citations were then screened based on the full publication to identify pertinent studies for inclusion. Disagreements were resolved through discussion until a consensus was reached or via the involvement of a third reviewer where necessary.

Inclusion and exclusion criteria are summarised in Table 1.

**Table 1:** Inclusion and exclusion criteria.

	Include	Exclude
Study design	Meta-analysis	Economic evaluations, cost studies, QoL studies, clinical trials, pooled analyses only and reviews/editorials
Disease/Population	T2DM	T1DM, any other disease and non-human studies
Intervention	Any pharmacological treatment for T2DM	Dietary supplements, dietary treatments and non-pharmacological management
Outcomes	Continuous HbA1c outcomes	No continuous HbA1c outcomes reported
Publication type	Full papers or abstracts	Protocol only
Language	English	Non-English language publications
Year of publication	2013 to present	Published before 2013

Abbreviations: QoL, quality of life; T1DM, type 1 diabetes mellitus; T2DM, type 2 diabetes mellitus

### Inclusion criteria

A publication was included if it detailed a standard pair-wise meta-analysis, indirect comparison or network meta-analysis of change in HbA1c in patients with T2DM, and it also assessed the use of a pharmacological intervention for treatment or management of T2DM. In order to obtain a reasonable study sample size of only the most recent studies to reflect the current practices, only English language publications published from 2013 onwards were considered.

### Exclusion criteria

Publications were excluded if the patient population did not have T2DM or the interventions assessed in the meta-analysis were dietary supplements or non-pharmacological management strategies, such as diet and exercise therapy.

### Data extraction

Data extraction was performed into an extraction template and verified by a second extractor. Disagreements were discussed with a third party.

## Results

**Fig 1:**
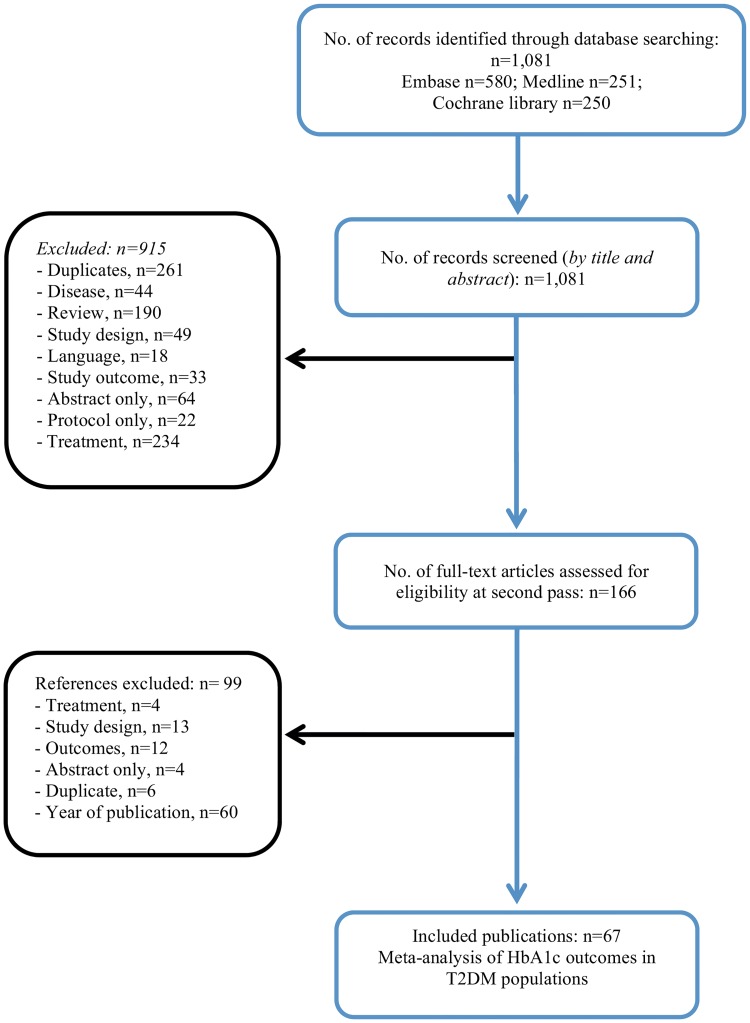
Study flow diagram.

A total of sixty-seven publications reporting a meta-analysis of change in HbA1c from baseline in T2DM patient populations were identified. The study flow diagram is shown in Fig 1.

A completed PRISMA checklist can be found in S2 Table. Thirty-nine (58%) of these publications did not report any approaches for dealing with missing variance data. Methods for handling missing variance data were reported in twenty-eight studies (42%) and are classified as algebraic recalculation, trial-level imputation, and no-imputation (Table 2).

**Table 2:** Summary of approaches to dealing with missing variance data across the included studies.

Approach	Method reported	Publication Count (%)
Not reported (n = 39)	No methods for handling missing data reported	39/67 (58)
Algebraic calculation	Computed from SE, 95% CIs or p-values	12/67 (18)
Trial-level imputation	Imputing SD using correlation coefficient of 0.5	4/67 (6)
	Imputing SD using correlation coefficient of 0.4	1/67 (1)
	Imputed values from a comparable study of the same treatment arms	1/67 (1)
	SDs were not reported, they were imputed by averaging reported SDs from other trials in the same comparison.	1/67 (1)
	SDs imputed using Cochrane methods	2/67 (3)
	Imputation from trial arms from the same drug class, using the prognostic method proposed by Ma et al., 2008 [6]	2/67 (3)
	Estimated by calculating the mean value of reported variances across main studies	1/67 (1)
	Data imputed but no methods reported	1/67 (1)
No imputation	Studies excluded from meta-analysis if insufficient information provided to enable SE calculation	4/67 (6)
Contacted authors	Attempts to contact author if data concerning outcome was missing	3/67 (5)

Abbreviations: CI, confidence interval; SD, standard deviation; SE, standard error

N.B a number of publications detailed both algebraic calculation and trial-level imputation

### Algebraic calculations

Twelve publications (18%) reported computation of SD from parametric summary statistics [11–22]. Six of these studies (9%) described the use of algebraic calculations but did not detail any further strategies for handling missing variance data [11–16]. A further six publications (9%) described the use of algebraic calculations and further strategies for imputation of missing SDs [17–22]. The following statistics were suggested for use across the publications: SEs, 95% CIs and p-values. None of the publications reported sufficient detail to assess which summary statistics were used in the calculations or to validate the calculations.

### Trial-level imputation

It is possible to calculate missing SDs with an imputed value of a correlation coefficient if baseline and final SDs are known [5]. Five studies (7%) reported imputing SDs using a correlation coefficient [18, 23–26]. The value used for the correlation coefficient may be imputed from another study, or alternatively a conservative estimate of 0.5 is recommended [1, 5]. Four of the studies (6%) reported that a correlation coefficient of 0.5 was used [18, 23, 25, 26]. A single study reported that a correlation coefficient of 0.4 was employed, which appeared to be obtained using data from previous meta-analyses, though this was not explicitly stated [24].

A single study imputed the SD from a comparable trial using the same treatment interventions [22]. Two studies (3%) reported imputing SDs by averaging across trials, although different approaches regarding study selection were implemented: i) averaging across all other ‘main’ studies [27] or ii) averaging across other trials of the same treatment comparisons [28].

Two studies (3%) [19, 21] reported the use of imputation methods outlined in the Cochrane handbook [5]. The potential imputation methods provided by Cochrane include using a correlation coefficient, borrowing values from one or more studies in the same meta-analysis or from another meta-analysis, or a more sophisticated method (such as linear regression) [8]. A single study [29] used a prognostic method which predicts missing SEs from known SEs using a previously published equation [6]. A single study which used a trial-level imputation approach to handle missing variance data reported a sensitivity analysis of the approximations by performing a no-imputation approach [28].

### No-imputation

Four studies (6%) excluded trials from their meta-analysis if there was insufficient information for algebraic calculation of SD [30–33]. None of the studies discussed the limitations and potential bias introduced by this approach in the publications.

Three studies reported attempts to contact authors for missing data but it was unclear if these attempts were successful and, if not, if other approaches (such as imputation) were explored [17, 34, 35].

### Sensitivity analyses

Three publications (4%) [23, 28, 36] detailed sensitivity analyses examining the impact of trial-level imputation approaches on analysis results; of these, one study did not clearly describe the sensitivity analyses performed or the results [36]. Another study fully reported that two sensitivity analyses were performed by assuming correlation coefficients of either 0.25 or 0.75, concluding that the different correlation coefficients did not change the results of the analysis [23]. A further study performed a sensitivity analysis which excluded the trials that did not report SD and concluded that the results of this analysis were broadly similar with the base case analysis [28].

## Discussion

A high proportion (58%) of recently reported meta-analysis publications in T2DM fail to report approaches to dealing with missing variance data for continuous outcomes. The majority of publications (42%) which do report a strategy for dealing with missing SDs are inconsistent or not comprehensive regarding the methods used for meta-analysis. The lack of transparency in approaches to handle missing variance data may in part be due to journal restrictions and a historical paucity regarding the standardisation and reporting of imputation methods.

Algebraic recalculation was stated in 18% of the identified meta-analysis publications, although it is expected that the large majority of the included studies will have used this approach given that the methods are outlined within the Cochrane handbook [5]. It was unclear if actual p-values were used in the algebraic calculations or if upper bound, critical, or non-significant p-values were employed in approximate algebraic recalculations. It is worth noting that such approximations are conservative and will result in larger SDs and a reduced weighting of that particular study in the meta-analysis [10].

The trial-level imputation methods identified in this review can be considered as relatively basic and included direct substitution, arithmetic mean substitution, and the imputation of correlation. There are other methods of imputation in the literature which were not applied in the identified studies. These methods include, but are not limited to, study level imputations from parametric summaries, regression with covariates, and coefficient of variation [10]. In addition, none of the methods identified in this review incorporated uncertainty associated with imputation. Multiple imputation is the only method which accounts for the implicit uncertainty from the missing SDs [9, 10, 37]. The findings of this study suggest that the choice of approach is based upon the simplicity and accessibility of methods with authors avoiding the use of other complex methods reported in the literature [9, 10, 37].

When missing SDs are imputed, some form of sensitivity analysis should be conducted to assess the robustness of the results of the analysis in relation to the assumptions made and as verification for the use of imputation. Few studies (4%) reported conducting sensitivity analyses surrounding the methods of imputation; those which did concluded that the results of the analyses were robust to the assumptions made. None of the studies explored sensitivity of the analyses to different methods of imputing the estimates of variance. However, three previous studies suggest that alternative methods for imputing estimates of variance do not alter the conclusions of a meta-analysis, although the generalisation of these findings to other meta-analyses is unknown [7, 38, 39].

As with all reviews, the current analysis was subject to limitations. A review is only as robust as the data supporting it; therefore, a main limitation of this research was poor reporting regarding the methods for handling missing variance data in the identified publications. Only English language publications were considered and there was no hand searching of grey literature. Only meta-analyses in T2DM were considered and the applicability of the results across other disease areas is unknown. However, we have no reason to believe the findings of this review wouldn’t be generalisable to meta-analyses of continuous outcomes, regardless of therapeutic area. The prevalence statistics obtained may be limited due to the relatively small sample of included studies (n = 67). Empirical work to compare methods to address missing variance data is beyond the scope of the review but further research to test the impact of the different approaches is recommended.

This study presented a review of the current methods used to address missing SDs in the meta-analysis of change in HbA1c in T2DM. The date restrictions of the literature searches have ensured that this review is representative of the most current practices used by authors to address missing variance data in meta-analysis. It is expected that this research represents the only published review of methods to deal with missing variance data focusing on a single therapeutic area. The use of a specific research question in a focused population facilitated data acquisition. The presented results are broadly consistent with the previous Wiebe et al. review, conducted over a decade ago, with searches performed in 2002 and no restriction regarding dates or the therapeutic area. The lack of standardisation of methods to address missing variance data appears to remain, although the current review identified a narrower range of methods used for variance imputation compared with those outlined in Wiebe at al. [10]. The brief guidelines on selecting a method for handling missing data set out by Wiebe at al. still represent a logical and systematic approach [10]. Further to this, authors are discouraged from using the no-imputation approach and are instead encouraged to improve the quality of reporting of their research. Sensitivity analyses are also encouraged and, as no method of imputation is known to be absolutely superior to another, exploration of different methods of imputation are desirable.

## Supporting Information

S1 Table Electronic literature searches. (DOCX)

S2 Table PRISMA checklist. (DOCX)
