# Supplementary material for: A Systematic Review of Methods for Handling Missing Variance Data in Meta-Analyses of Interventions in Type 2 Diabetes Mellitus
Source: PLoS One. 2016 Oct 17;11(10):e0164827. doi: 10.1371/journal.pone.0164827 (PMC5066955; doi:10.1371/journal.pone.0164827)
Supplement: S1 Table — (DOCX) [file pone.0164827.s001.docx]

**Embase** 1980 to 2015 Week 22

Searched 4th June 2015

| **#** | **Searches** | **Results** |
| --- | --- | --- |
| 1 | exp non insulin dependent diabetes mellitus/ | 157634 |
| 2 | ((type 2 or type-2 or type ii or type-ii or type two) adj2 diabet*).mp. | 134045 |
| 3 | (non insulin dependent diabetes mellitus or NIDDM or NDDM).mp. | 159905 |
| 4 | (T2DM or insulin independent diabet*).mp. | 15948 |
| 5 | maturity onset diabetes of the young.mp. | 1374 |
| 6 | ((late or adult* or matur* or slow or stable*) adj diabet*).mp. | 1813 |
| 7 | ((adult onset or adult-onset) and diabet*).mp. | 1209 |
| 8 | or/1-7 | 194824 |
| 9 | meta-analys*.mp. | 144019 |
| 10 | exp hemoglobin A1c/ | 53967 |
| 11 | ((glycated or glycosylated) adj1 h?emoglobin).mp. | 25015 |
| 12 | (HbA1c or Hb1c or A1c).mp. | 66207 |
| 13 | or/10-12 | 79273 |
| 14 | 8 and 9 and 13 | 1325 |
| 15 | limit 14 to yr="2013 -Current" | 580 |

**Ovid MEDLINE(R) In-Process & Other Non-Indexed Citations and Ovid MEDLINE(R)** 1946 to Present

Searched 4th June 2015

| **#** | **Searches** | **Results** |
| --- | --- | --- |
| 1 | exp Diabetes Mellitus, Type 2/ | 91988 |
| 2 | ((type 2 or type-2 or type ii or type-ii or type two) adj2 diabet*).mp. | 124889 |
| 3 | (non insulin dependent diabetes mellitus or NIDDM or NDDM).mp. | 9952 |
| 4 | (T2DM or insulin independent diabet*).mp. | 8499 |
| 5 | maturity onset diabetes of the young.mp. | 1030 |
| 6 | ((late or adult* or matur* or slow or stable*) adj diabet*).mp. | 1364 |
| 7 | ((adult onset or adult-onset) and diabet*).mp. | 962 |
| 8 | or/1-7 | 129035 |
| 9 | meta-analys*.mp. | 98761 |
| 10 | exp Hemoglobin A, Glycosylated/ | 24331 |
| 11 | ((glycated or glycosylated) adj1 h?emoglobin).mp. | 13263 |
| 12 | (HbA1c or Hb1c or A1c).mp. | 25713 |
| 13 | or/10-12 | 41817 |
| 14 | 8 and 9 and 13 | 607 |
| 15 | limit 14 to last 2 years | 251 |

CCTR, CDSR, DARE, CLHTA

Searched 4th June 2015

| **#** | **Searches** | **Results** |
| --- | --- | --- |
| 1 | exp Diabetes Mellitus, Type 2/ | 8097 |
| 2 | ((type 2 or type-2 or type ii or type-ii or type two) adj2 diabet*).mp. | 15132 |
| 3 | (non insulin dependent diabetes mellitus or NIDDM or NDDM).mp. | 5149 |
| 4 | (T2DM or insulin independent diabet*).mp. | 1525 |
| 5 | maturity onset diabetes of the young.mp. | 13 |
| 6 | ((late or adult* or matur* or slow or stable*) adj diabet*).mp. | 7246 |
| 7 | ((adult onset or adult-onset) and diabet*).mp. | 66 |
| 8 | or/1-7 | 19461 |
| 9 | meta-analys*.mp. | 30453 |
| 10 | exp Hemoglobin A, Glycosylated/ | 3474 |
| 11 | ((glycated or glycosylated) adj1 h?emoglobin).mp. | 5395 |
| 12 | (HbA1c or Hb1c or A1c).mp. | 6546 |
| 13 | or/10-12 | 9298 |
| 14 | 8 and 9 and 13 | 369 |
| 15 | limit 14 to last 2 years | 250 |
